# Competition between strains of *Borrelia afzelii* in the host tissues and consequences for transmission to ticks

**DOI:** 10.1038/s41396-021-00939-5

**Published:** 2021-03-03

**Authors:** Dolores Genné, Marika Rossel, Anouk Sarr, Florian Battilotti, Olivier Rais, Ryan O. M. Rego, Maarten J. Voordouw

**Affiliations:** 1 Laboratory of Ecology and Evolution of Parasites, Institute of Biology, University of Neuchâtel, Neuchâtel, Switzerland; 2 Laboratory of Ecology and Epidemiology of Parasites, Institute of Biology, University of Neuchâtel, Neuchâtel, Switzerland; 3 Biology Centre, Institute of Parasitology, Czech Academy of Sciences, České Budějovice, Czechia; 4 Faculty of Science, University of South Bohemia, České Budějovice, Czechia; 5 Department of Veterinary Microbiology, Western College of Veterinary Medicine, University of Saskatchewan, Saskatoon, Canada

**Keywords:** Microbial ecology, Bacteria

## Abstract

Pathogen species often consist of genetically distinct strains, which can establish mixed infections or coinfections in the host. In coinfections, interactions between pathogen strains can have important consequences for their transmission success. We used the tick-borne bacterium *Borrelia afzelii*, which is the most common cause of Lyme disease in Europe, as a model multi-strain pathogen to investigate the relationship between coinfection, competition between strains, and strain-specific transmission success. *Mus musculus* mice were infected with one or two strains of *B. afzelii*, strain transmission success was measured by feeding ticks on mice, and the distribution of each strain in six different mouse organs and the ticks was measured using qPCR. Coinfection and competition reduced the tissue infection prevalence of both strains and changed their bacterial abundance in some tissues. Coinfection and competition also reduced the transmission success of the *B. afzelii* strains from the infected hosts to feeding ticks. The ability of the *B. afzelii* strains to establish infection in the host tissues was strongly correlated with their transmission success to the tick vector. Our study demonstrates that coinfection and competition between pathogen strains inside the host tissues can have major consequences for their transmission success.

## Introduction

Most pathogen species are composed of different strains [1], which can vary in traits, such as infection intensity, virulence, and transmission [2–4]. This pathogen strain diversity can result in mixed infections or coinfections when hosts are infected with multiple strains [1, 2, 5]. Mixed infections can alter the fitness of the co-infecting strains compared to when they are alone in the host. However, the outcome of interactions between strains is highly variable and can range from facilitation to competition [5–7]. Furthermore, the performances of strains when they are alone in their host, are often not predictive of their performances when they are in a mixed infection [5, 8]. In summary, in systems where coinfections are common, interactions between strains may have important consequences for their transmission and hence the strain composition of the pathogen population.

Vector-borne pathogens are interesting systems for the study of mixed infections because pathogen strains can interact in both the vertebrate host [9–13] and the arthropod vector [14–17]. However, few studies have shown a direct relationship between strain-specific abundance in the tissues of co-infected hosts and strain-specific host-to-vector transmission success [3, 18]. In a rodent malaria system, the strain of *Plasmodium chabaudi* that established the highest abundance in the mouse blood was also the strain that had the highest mouse-to-mosquito transmission success [3]. Other vector-borne pathogens are found in tissues such as the skin, where the interactions between strains may be very different compared to a well-mixed liquid tissue such as the blood. Thus, studies of other systems will enhance our understanding of how inter-strain interactions in the vertebrate host shape strain-specific transmission success to the arthropod vector.

Our research group uses the tick-borne pathogen, *Borrelia afzelii*, as a model system to study interactions between strains in the rodent host and the tick vector. This spirochete bacterium belongs to the *Borrelia burgdorferi* sensu lato (sl) genospecies complex and is one of the most common causes of Lyme disease in Europe. *B. afzelii* is transmitted by the hard tick *Ixodes ricinus* and uses small mammals (e.g. rodents and shrews) as reservoir hosts [19]. *I. ricinus* consists of three different stages, larva, nymph, and adult, and each stage requires a blood meal to moult to the next stage, or to produce eggs in the case of adult females. Vertical transmission of spirochetes is rare [20], and larval *I. ricinus* ticks acquire *B. afzelii* during their first blood meal from an infected host. The skin, rather than the blood, is believed to be the critical organ for transmission of *B. afzelii* to feeding *I. ricinus* ticks [21–23].

*B. afzelii* contains dozens of genetically distinct strains [24, 25] and strain diversity is high at small spatial scales [9, 11, 26–30]. Both small mammal hosts [9, 11, 31] and *I. ricinus* ticks [26, 27] can be co-infected with multiple strains of *B. afzelii*. Previous studies on *B. burgdorferi* sl have found that coinfection in the vertebrate host results in competitive interactions that reduce the strain-specific abundance in host tissues [11] and the strain-specific host-to-tick transmission [14, 15, 32]. Suggested mechanisms of inter-strain competition include resource competition over limited host resources (e.g., nutrients, space) and apparent competition mediated by the host immune system [6, 7]. Surprisingly, there are no controlled studies for any *B. burgdorferi* sl genospecies that have investigated whether the effects of coinfection on strain-specific abundance in the host tissues influence strain-specific host-to-tick transmission success.

In this study, we used two strains of *B. afzelii* to investigate their interactions in a suitable rodent host, the laboratory mouse *Mus musculus*. For mice infected with single strains and for co-infected mice, we quantified the strain-specific presence and abundance in different organs and the strain-specific host-to-tick transmission success. We predict (1) that competition between strains will reduce the strain-specific presence and abundance in tissues of co-infected mice compared to mice with single strain infections; (2) that strains will exhibit preferences for different organs (strain-specific tissue tropism) and that the strength of inter-strain competition will differ among organs; (3) that competition between strains will reduce their transmission success from infected mice to feeding ticks; and (4) that the strain-specific presence and abundance in the host tissues will determine the strain-specific host-to-tick transmission success.

## Materials and methods

### Mice, ticks, and *Borrelia afzelii* strains

Forty female, 5-week-old, specific pathogen-free *Mus musculus* BALB/c mice were used in this study. We used BALB/c mice because we have a history of successful experimental infections with this mouse strain [33–35]. The *I. ricinus* ticks came from the laboratory colony that has been maintained at the University of Neuchâtel since 1978. We used *B. afzelii* strains NE4049 and Fin-Jyv-A3 in this study because we have a history of successful experimental infections with these two strains [33, 34, 36, 37]. Strain NE4049 was isolated from an *I. ricinus* tick in Switzerland, has multi-locus sequence type (MLST) 679, strain ID number 1887 in the *Borrelia* MLST database, and *ospC* major group (oMG) A10. Strain Fin-Jyv-A3 was isolated from a Finnish bank vole, has MSLT 676, strain ID number 1961 in the *Borrelia* MLST database and oMG A3. We determined that both strains contain all the plasmids necessary to complete the life cycle of *B. afzelii* (see section 1 in the electronic supplementary material (ESM)).

Our previous work has shown that *B. afzelii* can invade a diversity of tissues including the bladder, heart, ear, joints, and skin, and that the spirochete loads in internal organs (e.g., bladder and heart) are generally lower than external organs (ear and skin) [33, 36, 37]. In our previous work with strains Fin-Jyv-A3 and NE4049, we did not directly compare the tissue infection prevalence or tissue spirochete load between these two strains [36, 37], and we therefore have no a priori predictions with respect to these two phenotypes.

### Ethics approval

The commission that is part of the Service de la Consommation et des Affaires Vétérinaires (SCAV) of Canton Vaud, Switzerland evaluated and approved the ethics of this study. The veterinary service of the Canton of Neuchâtel, Switzerland issued the animal experimentation permit used in this study (NE04/2016).

### Experimental infection of mice via tick bite with one or two strains of *B. afzelii*

The study consisted of two independent experiments that differed with respect to the focal strain. In experiment 1, the focal strain was Fin-Jyv-A3; 10 mice were infected with strain Fin-Jyv-A3 (single infection) and 10 mice were simultaneously co-infected with strains Fin-Jyv-A3 and NE4049 (coinfection). In experiment 2, the focal strain was NE4049; 10 mice were infected with strain NE4049 (single infection) and 10 mice were simultaneously co-infected with strains NE4049 and Fin-Jyv-A3 (coinfection). Thus, across the two experiments, 10 mice were infected with strain Fin-Jyv-A3, 10 mice were infected with strain NE4049, and 20 mice were co-infected with both strains. All mice were infected via tick bite by using *I. ricinus* nymphs that had been experimentally infected with one of the two *B. afzelii* strains of interest (see section 2 in the ESM). Mice in the coinfection treatment were simultaneously infested with five nymphs infected with strain Fin-Jyv-A3 and five nymphs infected with strain NE4049. Mice in the single infection treatment were infested with five nymphs, infected with the focal strain and five uninfected nymphs, so that each mouse was infested with a total of 10 nymphs. To confirm *B. afzelii* infection, we took a blood sample at 4 weeks post-infection (PI) and used the SERION ELISA classic *Borrelia burgdorferi* IgG/IgM immunoassay to detect *Borrelia*-specific IgG antibodies.

### Host-to-tick transmission of *B. afzelii*

To measure host-to-tick transmission, mice were infested with *I. ricinus* larvae at 5 weeks PI as previously described (14, 15). The engorged larvae were allowed to moult into nymphs, which were sacrificed by freezing. For each mouse, a sample of 20 nymphs (range = 7–25) was tested for the two strains of *B. afzelii* as previously described (14, 15). For each mouse, the percentage of nymphs infected with the focal strain (Fin-Jyv-A3 or NE4049) is an estimate of the strain-specific transmission success.

### Mice dissection and DNA extraction

At 6 weeks PI, mice were euthanized with CO_2_. Mice were dissected, and six mouse organs were collected: bladder, left ear, right ear, heart, right ankle joint, and the section of dorsal skin where the nymphs had attached. For the left ear, right ear and dorsal skin, ~1 mg of tissue was sampled using a type II forceps (2 mm diameter). For the bladder, heart, and ankle joint, we used a scalpel to cut ~20 mg of tissue. DNA from the mouse tissue samples was extracted with Qiagen DNeasy Blood and Tissue kit 96-well plates and following the Qiagen protocol. Extracted DNA was eluted in 150 µl of autoclaved milliQ water.

### *Flagellin* qPCR

The total spirochete load in the mouse tissues was estimated using a qPCR that targeted a 132-bp fragment of the *flagellin* gene (33) using a previously described protocol (14) (see section 2 in the ESM). All samples were processed three times, each of the three replicates was performed in a different plate on a different day. All the plates contained three negative DNA extraction controls (tissues from uninfected mice), two negative controls for the qPCR (PCR-grade water), and four standards containing 10^2^, 10^3^, 10^4^, and 10^5^ copies of the *flagellin* gene. For the three independent qPCR assays, the overall repeatability of the Cq values was 86.9%. The mean of the Cq values of the three independent qPCRs was used to calculate the mean total spirochete load in 3 µl of DNA template for each of the mouse tissue samples.

### Nested strain-specific qPCRs

The two strains used in this study can be differentiated using the *ospC* gene; strains Fin-Jyv-A3 and NE4049 carry *ospC* major group alleles A3 and A10, respectively. The strain-specific spirochete load was estimated using two independent qPCR assays that targeted a 142-bp fragment of the *ospC* gene. However, because the spirochete load was very low in the mouse tissues, we first used a conventional PCR to enrich the number of *ospC* gene copies relative to the amount of mouse DNA. This PCR amplified a 657-bp fragment of the *ospC* gene using a previously described protocol [24] (see section 2 in the ESM). The amplicons from the conventional PCR were the template for the strain-specific *ospC* qPCR, and for this reason, the results from the latter were used to estimate the relative abundance (and not the absolute abundance) of the two strains in the tissue samples.

The amplicons from the conventional PCR were run in triplicate in the *ospC* A3 qPCR and in the *ospC* A10 qPCR to determine which strains were present. The primers that amplify the 142-bp fragment of the *ospC* gene are the same for the two strains; strain specificity is determined by the probes, which are different for each *ospC* allele. This strain-specific qPCR protocol has been described elsewhere [14] (see section 2 in the ESM).

### Repeatability of the strain-specific spirochete load

The repeatability of the strain-specific spirochete load was estimated by performing all three qPCR reactions in triplicate on the same set of 222 organ tissue samples. The overall repeatability (across all 222 organ tissue samples) of the spirochete load of strain Fin-Jyv-A3 and strain NE4049 was 78.9% and 87.0%, respectively (see section 2 in the ESM for details).

## Statistical Analysis

The statistical analyses were done using R version 1.2.5019 and the following packages: base, car, lme4 and emmeans. The *lmer()* and *glmer()* functions (lme4 package) were used to create the generalized linear mixed effects models (GLMMs) and linear mixed effects models (LMMs). The *Anova()* function (car package) and the *anova()* function (base package) were used to perform the log-likelihood ratio tests. The *emmeans()* and the *pairs()* functions (emmeans package) were used for the post-hoc analysis of the GLMMs and LMMs.

### Analysis of the *B. afzelii* tissue infection prevalence

The *flagellin* qPCR (which cannot distinguish between strains) was used to determine whether tissues were infected with *B. afzelii* or not. Tissue samples were considered infected with *B. afzelii* if at least two of the three replicate *flagellin* qPCR assays tested positive. The binomial response variable was whether *B. afzelii* was absent (0) or present (1) in a tissue sample, and it was analysed as a GLMM with binomial errors.

### Analysis of the total tissue spirochete load

The *flagellin* qPCR (which cannot distinguish between strains) was used to estimate the total *B. afzelii* spirochete load per mg of tissue in each mouse organ (or per mg of DNA). The spirochete loads estimated by the *flagellin* qPCR in 3 µl of DNA template were corrected to the total DNA extraction elution volume (150 µl), standardized by mg of tissue extracted (or by the DNA concentration of the DNA extraction), and log10 transformed to improve normality. The spirochete loads standardized by mg of tissue were highly correlated with the spirochete loads standardized by mg of DNA (*r* = *0.946*, *p* < 0.001; see section 3 in the ESM), and for brevity, only the former are shown in the main manuscript. The standardized log10-transformed tissue spirochete load was analysed as an LMM.

### Effect of strain, coinfection, and mouse organ on the strain-specific tissue infection prevalence

The focal strains in experiments 1 and 2 are Fin-Jyv-A3 and NE4049, respectively. In experiments 1 and 2, the tissue sample was considered infected with the focal strain (Fin-Jyv-A3 or NE4049) if at least two of the three replicate strain-specific qPCR assays (*ospC* type A3 or *ospC* type A10) tested positive. Thus, in experiments 1 and 2, the response variable is whether the mouse tissues tested positive for *ospC* type A3 versus *ospC* type A10, respectively, as determined by the two different strain-specific qPCR assays. The binomial response variable was whether the focal strain was absent (0) or present (1) in a tissue sample, and it was analysed as a GLMM with binomial errors. The fixed factors were focal strain (two levels: Fin-Jyv-A3, NE4049), coinfection (two levels: no, yes), mouse organ (six levels: bladder, left ear, right ear, heart, joint, and dorsal skin), and their interactions. Mouse identity was modeled as a random factor. Models that differed with respect to the fixed factor of interest were compared using log-likelihood ratio (LLR) tests to determine statistical significance.

### Effect of strain, coinfection, and mouse organ on the strain-specific tissue spirochete load

The focal strains in experiments 1 and 2 are Fin-Jyv-A3 and NE4049, respectively. For the co-infected mice, the abundance of each strain in a given tissue was estimated by combining the total spirochete abundance of the *flagellin* qPCR with the relative abundances of the *ospC* qPCRs (see section 2 in the ESM for details). Thus, in experiments 1 and 2, the response variable is the spirochete load of strain Fin-Jyv-A3 versus strain NE4049, respectively. These strain-specific spirochete loads were log10 transformed to improve normality and this variable was analysed using an LMM. The fixed factors, random factors, model simplification, and model analysis were the same as the analysis for the strain-specific tissue infection prevalence.

## Results

Eight mice were excluded from the study: two mice died during the experiment, one mouse was not infected, and five mice in the co-infected group only became infected with strain Fin-Jyv-A3. We believe that these five mice were not properly exposed to strain NE4049 (e.g., nymphs escaped from the capsule or refused to feed on the mice). If we include these five mice, our results become more significant because strain NE4049 is ‘excluded’ by strain Fin-Jyv-A3. The conservative approach is therefore to exclude these five mice. Thus, the final sample size consisted of 32 mice: strain Fin-Jyv-A3 alone (*n* = 9), strain Fin-Jyv-A3 in coinfection (*n* = 7), strain NE4049 alone (*n* = 9), and strain NE4049 in coinfection (*n* = 7).

### The *B. afzelii* infection prevalence differs among mouse organs

We estimated the prevalence of *B. afzelii* infection in the tissue samples of the six mouse organs (*n* = 192 tissue samples for 32 mice) using the *flagellin* qPCR. The rank order of tissue infection prevalence among the six mouse organs (from highest to lowest) was bladder (32/32 = 100.0%), left ear (29/32 = 90.6%), right ear (29/32 = 90.6%), heart (27/32 = 84.4%), ankle joint (28/32 = 87.5%), and dorsal skin (20/32 = 62.5%). The significant differences in tissue infection prevalence among organs (*p* < 0.001) indicate that *B. afzelii* exhibits tissue tropism (see section 4 in the ESM for a comprehensive analysis).

### The *B. afzelii* infection prevalence differs among strains

We tested whether the two *B. afzelii* strains differed in tissue tropism for the subset of mice infected with one strain (*n* = 18). We used a GLMM with binomial errors to analyse the tissue infection prevalence as a function of mouse organ and strain. The interaction between focal strain and organ was not significant (*p* = 0.286). The tissue infection prevalence for strain NE4049 (90.7% = 49/54) was significantly higher (1.2x) than that of strain Fin-Jyv-A3 (74.1% = 40/54; *p* = 0.015). Thus, strain NE4049 was more successful at establishing infection in rodent tissues than strain Fin-Jyv-A3. As shown previously, the tissue infection prevalence differed significantly among mouse organs (*p* = 0.024).

### The total tissue spirochete load of *B. afzelii* differs among mouse organs

**Fig. 1:**
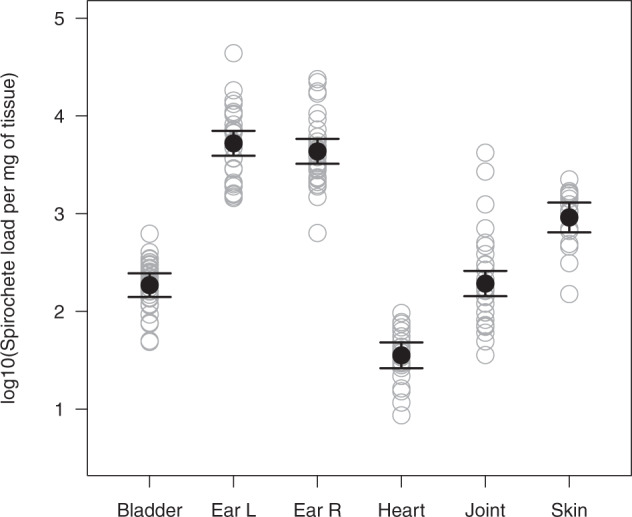
The spirochete load of *B*. ***afzelii*differs among the six mouse organs**. The tissue spirochete load was estimated using a *flagellin* qPCR and was standardized per mg of tissue. The log10-transformed spirochete load per mg of tissue is significantly different among the six mouse organs: bladder, left ear (Ear L), right ear (Ear R), heart, right ankle joint, and the section of dorsal skin where the nymphs had attached. Shown are the means, the 95% confidence intervals, and the individual data points.

We estimated the spirochete load of *B. afzelii* in the subset of infected mouse tissue samples (*n* = 165 tissue samples for 32 mice; uninfected tissues were excluded) using the *flagellin* qPCR. The heart, which had the lowest spirochete load, was set as the reference; the spirochete load was higher in the bladder (5.2x), ankle joint (5.4x), dorsal skin (25.7x), right ear (122.2x), and left ear (147.7x), and these differences among mouse organs were highly significant (Fig. 1; *p* < 0.000001; see section 5 in the ESM for the complete analysis). The results were similar when the spirochete loads were standardized per mg of DNA (see section 5 in the ESM). The matrix of pairwise correlations in the total spirochete load between the six mouse organs found that the highest correlations were for pairs of skin-related organs (see section 6 in the ESM).

### The total tissue spirochete load differs among strains

We tested whether the two *B. afzelii* strains differed in tissue bacterial abundance for the subset of mice infected with one strain (*n* = 89 tissue samples for *n* = 18 mice; uninfected tissues were excluded). We used an LMM to analyse the standardized log10-transformed strain-specific tissue spirochete loads as a function of mouse organ and focal strain. The interaction between focal strain and mouse organ was significant (*p* = 0.037), and we therefore compared the tissue spirochete load between the two strains separately for each mouse organ. The mean spirochete load in the ankle joints for strain Fin-Jyv-A3 (mean = 347; 95% CI = 184–655; units = spirochetes per mg of tissue) was significantly higher (3.8x) than that of strain NE4049 (mean = 98; 95% CI = 61–158; *p* = 0.0016). There were no significant differences in spirochete load between the two strains for the other five mouse organs (see section 7 in the ESM). Thus, strain Fin-Jyv-A3 established higher spirochete loads in the joint tissues than strain NE4049.

### Effect of strain, coinfection, and mouse organ on the strain-specific tissue infection prevalence

**Fig. 2:**
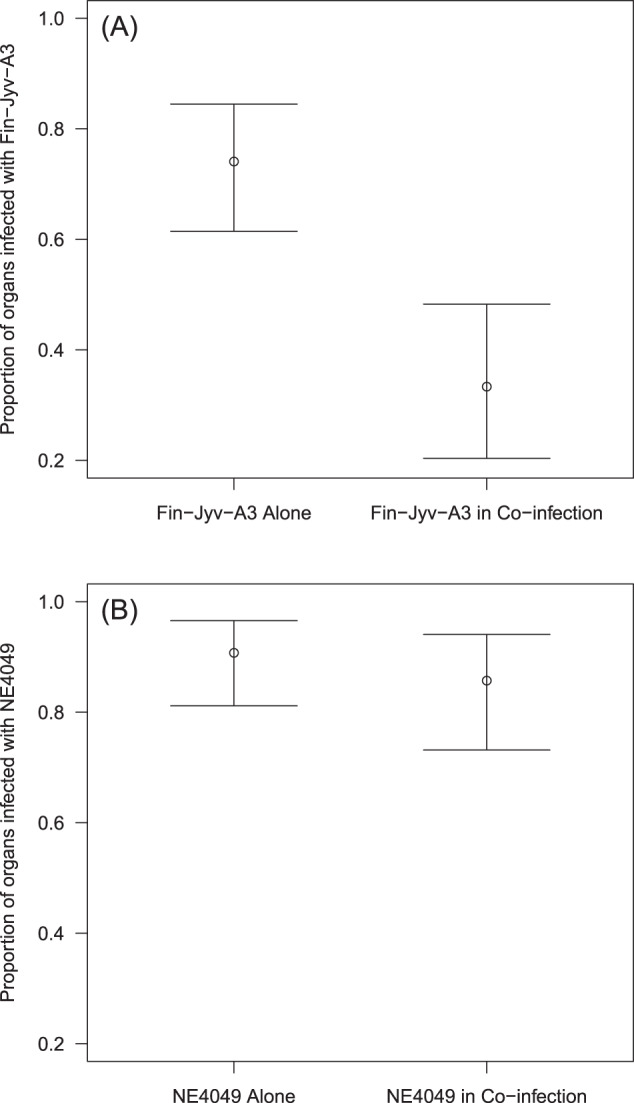
Coinfection in the rodent host influences the proportion of mouse organs infected by the focal strain. Panels **A** and **B** show the results from two independent experiments where the focal strains are Fin-Jyv-A3 and NE4049, respectively. The interaction between focal strain and coinfection was almost significant (*p* = 0.080). When the strains were analysed separately, coinfection reduced the strain-specific prevalence significantly for (**A**) strain Fin-Jyv-A3 (*p* < 0.0001) but not for (**B**) strain NE4049 (*p* = 0.445). Shown are the means and the 95% confidence intervals.

The strain-specific tissue infection prevalence refers to whether a tissue was infected with the focal strain. We used a GLMM with binomial errors to analyse the strain-specific infection prevalence in the mouse tissues as a function of focal strain, coinfection and mouse organ (see section 8 in the ESM for the complete analysis). Non-significant terms were removed using stepwise model simplification. The interaction between strain and coinfection was almost significant (Fig. 2; *p* = 0.080) and we therefore split the analysis by strain. Coinfection in the rodent host significantly reduced the number of mouse organs infected with strain Fin-Jyv-A3 (Fig. 2; *p* = 0.00007). In contrast, coinfection in the rodent host had no effect on the number of mouse organs infected with strain NE4049 (Fig. 2; *p* = 0.446). Thus, coinfection resulted in competition between strains, which reduced the presence of strain Fin-Jyv-A3, but not strain NE4049, in the host tissues (see section 9 in the ESM for an additional analysis).

### Effect of strain, coinfection, and mouse organ on the strain-specific tissue spirochete load

For the subset of infected mouse tissue samples (*n* = 165), we used an LMM to analyse the strain-specific spirochete load in the mouse tissue samples as a function of focal strain, coinfection and mouse organ (see section 10 in the ESM for the complete analysis). A classic stepwise model simplification approach found that the three-way interaction was significant (*p* = 0.031), and the analysis was therefore divided by focal strain. For strain Fin-Jyv-A3, the interaction between coinfection and mouse organ was significant (*p* = 0.0002); we therefore tested the effect of coinfection separately for each mouse organ. In the bladder, coinfection significantly decreased (4.0x) the mean spirochete load of strain Fin-Jyv-A3 (*p* = 0.003). In the ankle joint, coinfection significantly increased (5.9x) the mean spirochete load of strain Fin-Jyv-A3 (*p* = 0.022). The contrast between single infection and coinfection was not significant for the other three mouse organs. For strain NE4049, the interaction between coinfection and mouse organ was not significant (*p* = 0.677). Coinfection was not significant (*p* = 0.061), but as before, mouse organ had a significant effect on the strain-specific tissue spirochete load (*p* < 0.000001). The results were similar when the tissue spirochete loads were standardized per mg of DNA (see section 11 in the ESM).

### Effects of coinfection and strain on host-to-tick transmission of the focal strain

**Fig. 3:**
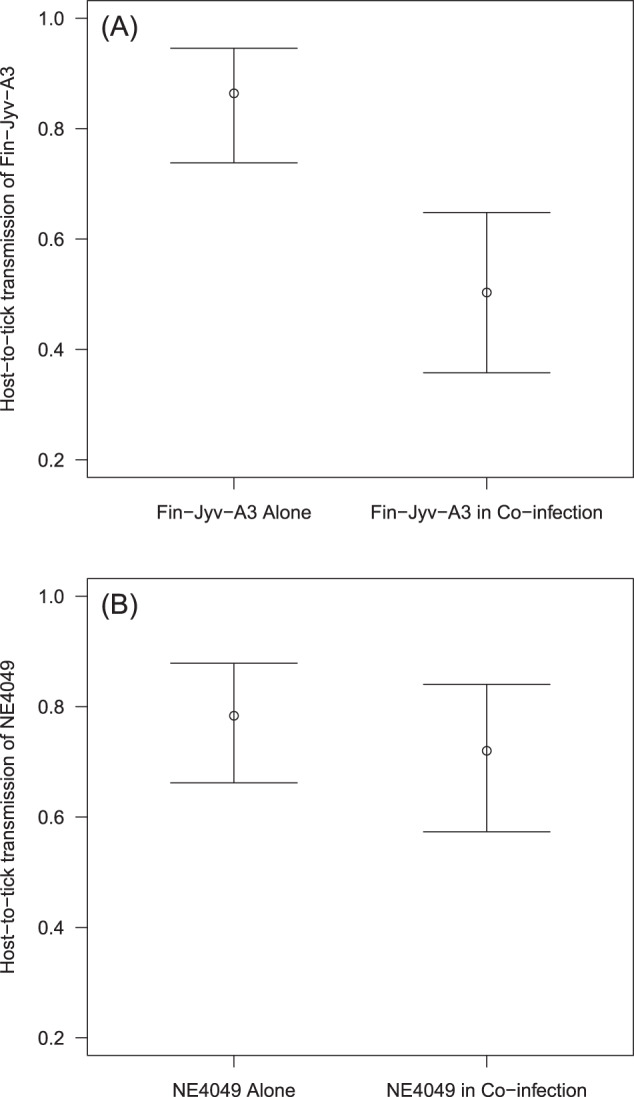
Coinfection in the rodent host influences the host-to-tick transmission of the focal strain. Panels **A** and **B** show the results from two independent experiments where the focal strains are Fin-Jyv-A3 and NE4049, respectively. The interaction between focal strain and coinfection was significant (*p* = 0.028), and we therefore split the analysis by strain. Coinfection reduced host-to tick transmission for both strains; the effect was significant for (**A**) strain Fin-Jyv-A3 (*p* = 0.0006), but not for (**B**) strain NE4049 (*p* = 0.492). Shown are the means and the 95% confidence intervals.

We used a GLMM with binomial errors to test whether strain and coinfection influenced the strain-specific host-to-tick transmission success. The interaction between strain and coinfection was significant (Fig. 3; *p* = 0.028), and we therefore split the analysis by strain. For strain Fin-Jyv-A3, coinfection significantly reduced host-to-tick transmission (Fig. 3; *p* = 0.0006). In contrast, for strain NE4049, coinfection had no effect on host-to-tick transmission (Fig. 3; *p* = 0.492).

### Relationship between host-to-tick transmission of the focal strain and the presence and abundance of the focal strain in the mouse organs

We used a GLMM with binomial errors to test whether variation in the strain-specific host-to-tick transmission success depended on either (1) the number of mouse organs infected with the focal strain, or (2) the mean spirochete load of the focal strain for the subset of infected mouse organs.

**Fig. 4:**
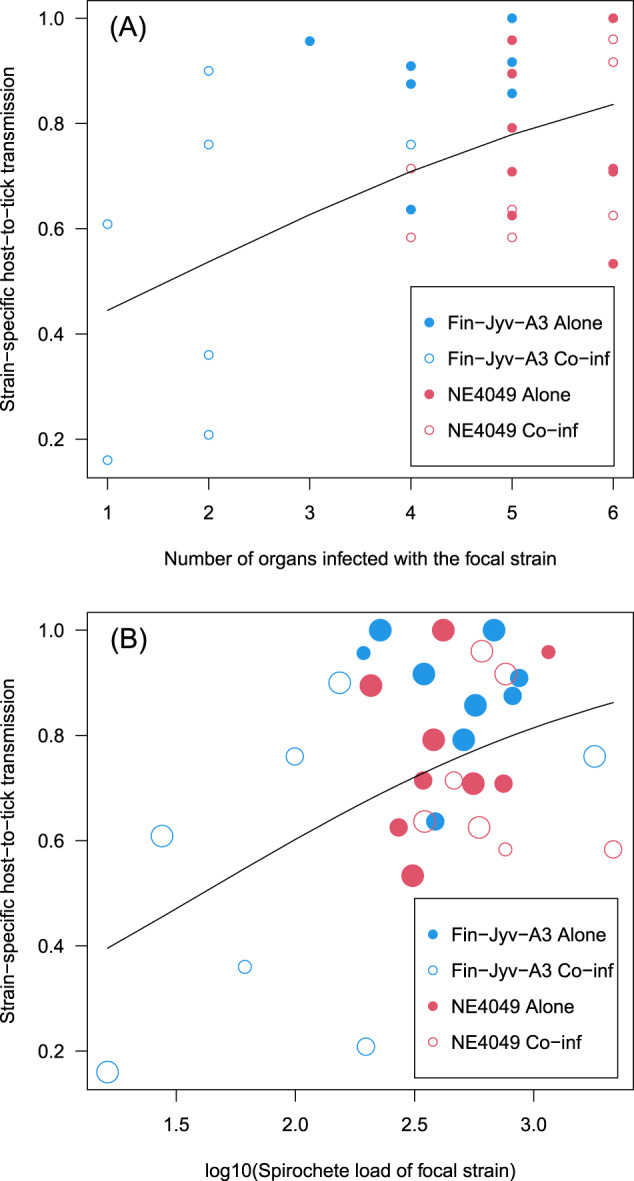
Host-to-tick transmission of the focal strain depends on the number of mouse organs that are infected with the focal strain or the mean spirochete load of the focal strain in the mouse organs. (**A**) The six mouse organs included the bladder, left ear, right ear, heart, right ankle joint, and the section of dorsal skin where the nymphs had attached. (**B**) The log10-transformed spirochete load of the focal strain was averaged over the subset of infected organs (the size of each circle indicates the number of infected organs). Strain Fin-Jyv-A3 and strain NE4049 are shown in blue and red colours, respectively. Mice in the single strain groups and the co-infected groups are shown with solid and empty symbols, respectively. The black line shows the line of best fit from a generalized linear model (GLM) with binomial errors.

The positive relationship between the number of mouse organs infected with the focal strain and host-to-tick transmission of the focal strain was highly significant (Fig. 4(A); *p* = 0.0005; see section 12 in the ESM). Similarly, the positive relationship between the mean spirochete load of the focal strain for the subset of infected mouse organs and host-to-tick transmission of the focal strain was highly significant (Fig. 4(B); *p* = 0.003; see section 13 in the ESM). After correcting for differences among organs, the positive relationship between the mean spirochete load of the focal strain for the subset of infected organs and host-to-tick transmission of the focal strain was almost significant (*p* = 0.050; see section 13 in the ESM).

## Discussion

### Prevalence of *B. afzelii* in the tissues of the rodent host

Overall, the prevalence of *B. afzelii* infection in the mouse organs was high; five of the six mouse organs had an infection prevalence >80.0%. These results were similar to a recent experimental infection study with strain NE4049 in the same host (27 female BALB/c mice) where 92.6% of the tissue samples (100/108) tested positive for *B. afzelii* (34). In the present study, we found that NE4049 established infection in more tissues than Fin-Jyv-A3, which suggests that NE4049 is more invasive or more infectious than Fin-Jyv-A3.

### Spirochete load of *B. afzelii* in the tissues of the rodent host

The spirochete load of *B. afzelii* per mg of tissue differed greatly between the mouse organs. For the subset of infected tissues, we found that the spirochete load was highest in the ears and the dorsal skin, and lower in internal organs like the heart, bladder, and ankle joints. Other studies on *B. afzelii* and *B. burgdorferi* sensu stricto (ss) in rodent hosts, have also found that the spirochete load is higher in the skin compared to internal organs like the bladder and heart [33, 36–39]. One explanation for this result is that the skin is important for the host-to-tick transmission of *B. burgdorferi* sl [21–23], whereas the internal organs (e.g., bladder, heart, and joints) are a dead-end for spirochete transmission to ticks (39).

For the mice that were infected with single strains, we found that strain Fin-Jyv-A3 established a higher spirochete load in the ankle joints compared to strain NE4049. Previous studies on *B. afzelii* and *B. burgdorferi* ss in lab mice have shown differences in tissue spirochete load between strains [21, 38, 40, 41]. This strain-specific variation is important from a medical perspective because it has been associated with host pathology including carditis, arthritis, and ankle joint swelling [40, 41]. The higher abundance of Fin-Jyv-A3 in the joints suggests that this strain would cause more arthritis and joint swelling in the host compared to NE4049, as observed for strains of *B. burgdorferi* ss in lab mice [40, 41].

### Competition between strains of *B. afzelii* in the tissues of the rodent host

Our study demonstrated that coinfection of mice with two strains of *B. afzelii* resulted in competitive interactions between strains in the host tissues. We measured the outcome of coinfection in two ways: (1) strain-specific tissue infection prevalence and (2) strain-specific tissue spirochete load. For strain Fin-Jyv-A3, coinfection reduced the tissue infection prevalence by 55%, indicating a strong effect of competition. The results of our study agree with other studies on vector-borne pathogens that have shown that coinfection leads to competitive interactions in the vertebrate host [3, 10, 42]. Our study is the first experimental demonstration that strains belonging to the same *B. burgdorferi* sl genospecies compete inside the tissues of the vertebrate host. An uncontrolled field study in Sweden on small mammals naturally infected with *B. afzelii* previously found results consistent with inter-strain competition in the host [11]. Other studies on *B. afzelii* and *B. burgdorferi* ss have shown that coinfection in the rodent host reduced strain-specific transmission to ticks [14, 15, 32, 43], however, this result could be caused by interactions in the rodent host, the tick vector, or both. In contrast, our controlled study clearly shows that coinfection and competitive interactions reduce the ability of some *B. afzelii* strains to establish infection in the tissues of the rodent host.

With respect to tissue spirochete load, we found that the outcome of the interactions between strains depended on the mouse organ. For strain Fin-Jyv-A3, coinfection in the mouse host had opposite effects on the bacterial abundance in the bladder (decrease) and ankle joints (increase). We had expected that inter-strain competition would be most intense in the ears and dorsal skin because these organs are critical for host-to-tick transmission, but this was not the case. Our study shows the importance of sampling numerous organs when testing whether coinfection influences the strain-specific abundance in the tissues of the vertebrate host.

Our study does not elucidate the mechanism of competition, which includes interference, exploitation, and apparent competition [6, 7, 44]. One plausible mechanism is exploitation competition, which occurs when pathogen strains compete over limited host resources such as nutrients or space [42, 45, 46]. Another plausible mechanism is apparent competition, where the host immune system mediates the interactions between strains [47, 48]. Interference competition is unlikely because this mechanism requires the production of toxic substances that weaken the performance of other strains, but *B. burgdorferi* sl does not produce such toxins [49].

### Pathogen distribution in host tissues and host-to-vector transmission

Host-to-vector transmission is one of the critical life history traits for any vector-borne pathogen and determines the frequency of a strain in nature and whether it is common or rare [28, 35, 50]. As we have shown previously [14, 15], coinfection in the rodent host and competition between strains reduced the host-to-tick transmission of the focal strain. What is novel about this study is our demonstration that inter-strain competition reduced the presence of the two strains in the rodent tissues, which in turn, reduced their host-to-tick transmission success. Further evidence for the link between tissue pathogen loads and transmission was our demonstration that both the mean tissue infection prevalence and the mean tissue spirochete load were positively related to host-to-tick transmission of the focal strain. Our study agrees with studies on the rodent malaria parasite (*P. chabaudi*) in lab mice, where strains with higher abundance in the blood have higher mouse-to-mosquito transmission [3, 18, 51, 52]. Previous studies on *B. afzelii* found a positive relationship between the spirochete load in rodent ear tissues and host-to-tick transmission [21, 22], but these studies did not investigate inter-strain competition. Our study is the first experimental demonstration that competitive interactions between strains of *B. burgdorferi* sl in the host tissues determine strain-specific host-to-tick transmission success.

### Inter-strain competition and strain diversity of *B. burgdorferi* sl pathogens in nature

In nature, there is substantial strain diversity of *B. burgdorferi* sl pathogens at small spatial scales [26, 29, 53, 54]. Studies have shown that some strains are much more common than others, and that these differences in strain frequency are constant over time and space [28, 30, 55, 56]. A major question is to understand the ecological factors that maintain the strain composition of the pathogen population. The multiple niche polymorphism (MNP) hypothesis proposes that the different strains are adapted to different vertebrate reservoir hosts [53, 57]. The present study suggests that competitive interactions between strains in the host tissues can also have important consequences for their host-to-tick transmission. The MNP hypothesis and the inter-strain competition hypothesis are not mutually exclusive; for example, the competitive ability of strains could differ between vertebrate hosts. Future studies should investigate the relative importance of MNP versus inter-strain competition in maintaining the strain diversity of *B. burgdorferi* sl pathogens in nature.

### Conclusions

For multi-strain vector-borne pathogens, we show that competition between strains in the host tissues can reduce host-to-vector transmission. Simultaneous coinfection of the rodent host can inhibit the ability of strains to invade and establish infection in the host tissues. Depending on the organ, coinfection and interactions between strains either increased or decreased the spirochete abundance of a given strain. The ability to establish infection in many tissues and the mean bacterial abundance were both positively related with strain-specific host-to-tick transmission success.

## Supplementary information

Supplemental material
