# Supplementary material for: Competition between strains of Borrelia afzelii in the host tissues and consequences for transmission to ticks
Source: ISME J. 2021 Mar 3;15(8):2390–400. doi: 10.1038/s41396-021-00939-5 (PMC8319436; doi:10.1038/s41396-021-00939-5)
Supplement: Supplementary file 1 — Supplemental material [file 41396_2021_939_MOESM1_ESM.docx]

Supplementary material

Title: Competition between strains of *Borrelia afzelii* in the host tissues and consequences for transmission to ticks

Authors: Dolores Genné, Marika Rossel, Anouk Sarr, Florian Battilotti, Olivier Rais, Ryan O. M. Rego, and Maarten J. Voordouw

**Table of Contents**

[Section 1 – The plasmid profile of *B. afzelii* strains Fin-Jyv-A3 and NE4049 2](#_Toc63287094)

[Section 2 – Experimental methods and molecular methods 3](#_Toc63287095)

[Section 3 – Analysis of the total spirochete load 5](#_Toc63287096)

[Section 4 – The *B. afzelii* tissue infection prevalence differs among organs 5](#_Toc63287097)

[Section 5 – Analysis of the total spirochete load of *B. afzelii* 6](#_Toc63287098)

[Section 6 – Correlations in spirochete loads among mouse organs 10](#_Toc63287099)

[Section 7 – The total tissue spirochete load differs among strains 14](#_Toc63287100)

[Section 8 – Effect of strain, co-infection, and organ on the strain-specific tissue infection prevalence 14](#_Toc63287101)

[Section 9 – Effect of co-infection on the strain-specific tissue infection prevalence in the mice 16](#_Toc63287102)

[Section 10 – Effect of strain, co-infection, and organ on the strain-specific tissue spirochete load per mg of tissue 17](#_Toc63287103)

[Section 11 – Effect of strain, co-infection, and organ on the strain-specific tissue spirochete load per mg of DNA 21](#_Toc63287104)

[Section 12 – Relationship between host-to-tick transmission of the focal strain and the number of organs infected by the focal strain 23](#_Toc63287105)

[Section 13 – Relationship between host-to-tick transmission of the focal strain and the mean organ spirochete load of the focal strain 24](#_Toc63287106)

[Section 14 – References 27](#_Toc63287107)

# Section 1 – The plasmid profile of *B. afzelii* strains Fin-Jyv-A3 and NE4049

We used PCR to determine whether the two strains used in this study, Fin-Jyv-A3 and NE4049, contained all the plasmids necessary to complete the life cycle of *B. afzelii*. We tested the strains for the following 16 plasmids: lp 17, lp 28-2, lp 28-3, lp 25, lp 28, lp 28-4, lp 28-7, lp 28-8, lp 30, lp 34, lp 38, cp 26, cp 32-5, cp 32-7, cp 32-9, cp 32-10. Total genomic DNA was prepared from approximately 8 ml of *Borrelia* culture using the Wizard genomic DNA kit (Promega, Madison, WI) as per the manufacturer’s instructions. The total plasmid contents of both strains of *B. afzelii* were compared using primers specific for plasmids based on the complete genome sequence of PKo and ACA-1 (1). In addition, primers specific for the genes *pncA* and *adeC* were used to confirm their presence (1).

The plasmid profile of *B. afzelii* strains Fin-Jyv-A3 and NE4049 are compared to those of *B. afzelii* strains PKo and CB43 (Table S1). Of the 16 tested plasmids, strains Fin-Jyv-A3 and NE4049 contained 15 and 14 plasmids, respectively. Both strains were missing cp 32-7, which was present in both strains PKo and CB43. Strain NE4049 was also missing cp 32-5, which was also missing in strain CB43, but this plasmid was present in strains Fin-Jyv-A3 and PKo. In summary, the two strains that we used in this study, Fin-Jyv-A3 and NE4049, contained all the plasmids that are necessary to complete the life cycle of *B. afzelii*.

Table S1. The plasmid profile is shown for four strains of *B. afzelii*: PKo, CB43, Fin-Jyv-A3 and NE4049. The 16 plasmids tested were as follows: lp 17, lp 28-2, lp 28-3, lp 25, lp 28, lp 28-4, lp 28-7, lp 28-8, lp 30, lp 34, lp 38, cp 26, cp 32-5, cp 32-7, cp 32-9, cp 32-10. The ‘+’ and ‘-’ symbols indicate whether a plasmid was present or absent for a given strain. Some of the plasmids were tested for with different primer sets.

| Plasmids | PKo | CB43 | Fin-Jyv-A3 | NE4049 |
| --- | --- | --- | --- | --- |
| lp 17 | + | + | - | + |
| ACA-1 lp 17 | + | + | + | + |
| lp 28-2 | + | + | - | + |
| ACA-1 lp 28-2 | + | + | + | + |
| lp 28-3 | + | + | - | + |
| ACA-1 lp 28-3 | + | + | + | + |
| lp 25 | + | + | + | + |
| lp 28 | + | + | + | + |
| lp 28-4 | + | + | + | + |
| lp 28-7 | + | + | + | + |
| lp 28-8 | + | + | + | + |
| lp 30 | + | + | + | + |
| lp 34 | + | + | + | + |
| lp 38 | + | + | + | + |
| cp26 | + | + | + | + |
| cp 32-5 | + | - | + | - |
| cp 32-7 | + | + | - | - |
| cp 32-9 | + | + | + | + |
| cp 32-10 | + | + | + | + |

# Section 2 – Experimental methods and molecular methods

**Creation of infected nymphs:** Female BALB/c mice were infected with either strain Fin-Jyv-A3 or strain NE4049 via needle inoculation. *I. ricinus* larvae were fed on the infected mice at 4 weeks post-infection (PI) and the engorged larvae were collected and kept individually in Eppendorf tubes. The Eppendorf tubes contained a piece of moistened paper towel to increase the relative humidity and ensure high larva-to-nymph moulting success. After the larva-to-nymph moult, we randomly selected a sample of nymphs to determine the prevalence of *B. afzelii* infection. For strain Fin-Jyv-A3 and strain NE4049, the prevalence of infection in the nymphs was 70.0% (7/10) and 71.4% (10/14), respectively.

**Host-to-tick transmission of *B. afzelii*:** To measure host-to-tick transmission, mice were infested with larval ticks at 5 weeks PI as previously described (2, 3). The engorged larval ticks were allowed to moult into nymphs, which were sacrificed by freezing at either 1 month or 4 months after the larva-to-nymph moult. For each mouse, 10 one-month-old nymphs and up to 15 four-month-old nymphs (mean of tested nymphs per mouse = 20.3, range = 7 – 25) were analysed for *B. afzelii* infection as previously described (2, 3). Thus, for each mouse, we obtained two estimates of host-to-tick transmission; one estimate was based on 10 one-month-old nymphs, the other estimate was based on up to 15 four-month-old nymphs. We had previously shown that the age at which the nymph was frozen (1 month or 4 months) does not affect the infection prevalence of the two strains of *B. afzelii* (3). We are therefore justified in combining these two groups of nymphs to obtain a single and best estimate of host-to-tick transmission success.

**Correlation in host-to-tick transmission between the 1-month-old nymphs and the 4-month-old nymphs:** To measure host-to-tick transmission, each mouse was infested with larval ticks that were allowed to moult into nymphs that were frozen at either 1 month or 4 months of age. Host-to-tick transmission of the focal strain was measured as the proportion of 1-month-old nymphs and 4-month-old nymphs infected with the focal strain. We found a significant positive correlation in the host-to-tick transmission of the focal strain between the 1-month-old nymphs and the 4-month-old nymphs (*r = 0.745*, df = 28, t = 5.901, *p < 0.00001*). The two groups of nymphs were therefore combined to give a single and best estimate of host-to-tick transmission success.

***Flagellin* qPCR:** The total spirochete load in the mouse tissues was estimated using a qPCR that targeted a 132-bp fragment of the *flagellin* gene (4). The qPCRs were performed using the LightCycler^®^ 480 Multiwell Plate 96 white (Roche). The wells were filled with a mixture of 5.8 µl of water, 10.0 µl of Master Mix (FastStart Essential DNA probes Master, Roche), 0.4 µl of 20.0 µM forward primer FlaF1A, 0.4 µl of 20.0 µM reverse primer FlaR1, 0.4 µl of 10.0 µM Flaprobe1, and 3.0 µl of DNA template. The thermocycling conditions consisted of 10 min at 95°C for denaturation, followed by 50 cycles of 30 sec at 60°C and 10 sec at 95°C.

**Conventional PCR to amplify the *ospC* gene:** The spirochete load was very low in the mouse tissues, and we therefore used a conventional PCR to enrich the number of *ospC* gene copies relative to the amount of mouse DNA. This PCR amplified a 657-bp fragment of the *ospC* gene using a previously described protocol (5). The forward and reverse primers were 5’-ATGAAAAAGAATACATTAAGTGC-3’ (positions 306–328 of U01894) and 5’-ATTAATCTTATAATATTGATTTTAATTAAGG-3’ (positions 963–933 of U01894), respectively. The mixture contained 14.8 µl of water, 5.0 µl of Mastermix, 1.5 µl of 10.0 mM forward primer, 1.5 µl of 10.0 mM reverse primer, 0.2 µl of Taq, and 2.0 µl of DNA template. The thermocycling program was 94°C for 3 min, 35 cycles of [94°C for 45 s, 52°C for 45 s, and 72°C for 45 s], 72°C for 7 min, and cooling at 10°C. Each plate contained 2 wells filled with the reagents and water and 3 wells that had DNA from uninfected mice as template. The amplicons from the conventional PCR were the template for the strain-specific *ospC* qPCR.

**Strain-specific qPCRs targetting the *ospC* gene:** The amplicons from the conventional PCR were run in triplicate in the *ospC* A3 qPCR and in the *ospC* A10 qPCR to determine which strains were present. The primers are the same for the two strains and amplify a 142-bp fragment of the *ospC* gene. Strain specificity is determined by the probes, which are different for each *ospC* allele. The qPCRs were performed using the LightCycler® 480 Multiwell Plate 96 white (Roche). The wells were filled with 10.0 μl of 2× Master Mix (FastStart Essential DNA Probes Master, Roche Applied Science), 5.8 μl of water, 0.4 μl of 20.0 mM forward primer OspC_qPCR_For, 0.4 μl of 20.0 mM reverse primer OspC_qPCR_Rev, 0.4 μl of 10.0 mM A3_Probe or A10_Probe, and 3.0 μl of DNA template (2). Each plate contained 5 wells filled with the negative controls from the PCR and 2 wells filled with the reagents of the qPCR and water.

**Repeatability of the strain-specific spirochete load:** The repeatability of the strain-specific spirochete load was calculated as follows. The total spirochete abundance in the mouse tissues was estimated using the three independent *flagellin* qPCRs. Similarly, the relative abundance of strains Fin-Jyv-A3 and NE4049 was calculated using the three independent *ospC* A3 qPCRs and the three independent *ospC* A10 qPCRs. For each strain, the three estimates of total spirochete load were paired with the three estimates of relative abundance to calculate three estimates of the strain-specific spirochete load for each of the 222 organ tissue samples (37 mice* 6 organs per mouse = 222 organ tissue samples). The overall repeatability (across all 222 organ tissue samples) of the spirochete load of strain Fin-Jyv-A3 and strain NE4049 was 78.87% and 87.04%, respectively. All six *ospC* qPCRs were done on the same set of *ospC* amplicons, so our estimate of the repeatability does not include variation due to differences among amplicons coming from different conventional PCR reactions.

**Estimation of the strain-specific spirochete load:** The focal strains in experiments 1 and 2 are Fin-Jyv-A3 and NE4049, respectively. For the co-infected mice, the abundance of each strain in a given tissue was estimated by combining the total spirochete abundance of the *flagellin* qPCR with the relative abundances of the *ospC* qPCRs. For example, if the total spirochete load for a given tissue sample was 200 spirochetes per mg of tissue, and the relative abundances of strains Fin-Jyv-A3 and NE4049 were 75% and 25% respectively, then the strain-specific abundances of strains Fin-Jyv-A3 and NE4049 were 150 and 50 spirochetes per mg of tissue, respectively. For each of the 222 tissue samples, we had 3 estimates of total spirochete load and 3 estimates of the relative abundance, which allowed us to estimate 3 independent estimates of the strain-specific spirochete load. We calculated an average strain-specific spirochete load for each of the 222 tissue samples by taking the geometric mean of the 3 independent estimates. These averaged strain-specific spirochete loads were used in the statistical analyses of the strain-specific spirochete loads.

# Section 3 – Analysis of the total spirochete load

The spirochete loads analysed in this study were standardized by mg of tissue extracted. When the spirochete loads were standardized by mg of DNA and compared to the spirochete loads standardized by mg of tissue, the correlation was positive and highly significant (Figure S1; *r = 0.946*, df = 220, t = 43.11, *p < 0.000001*).


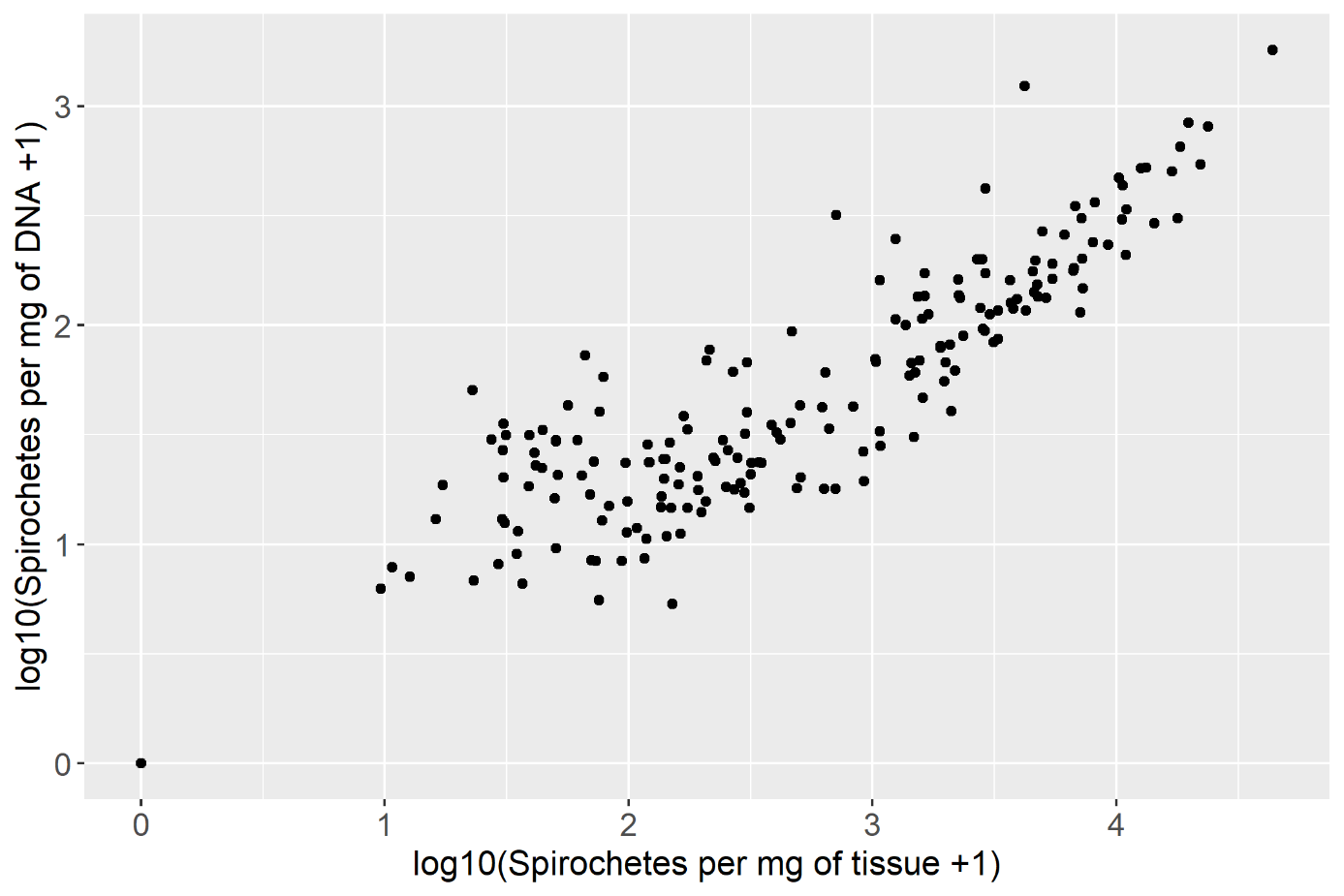


Figure S1. The two methods of standardizing the spirochete load in the mouse tissues give very similar results. The tissue spirochete loads were expressed as the spirochetes per mg of tissue (horizontal axis) or the spirochetes per mg of DNA (vertical axis).

# Section 4 – The *B. afzelii* tissue infection prevalence differs among organs

The *flagellin* qPCR, which cannot distinguish between strains, was used to determine whether tissues were infected with *B. afzelii* or not. Tissue samples were considered infected with *B. afzelii* if at least two of the three replicate *flagellin* qPCR assays tested positive. The sample size was 32 mice with 6 tissues per mouse for a total of 192 tissue samples. The binomial response variable was whether *B. afzelii* was absent (0) or present (1) in a tissue sample, and it was analysed as a generalized linear mixed effects model (GLMM) with binomial errors. The fixed factors were focal strain (two levels: Fin-Jyv-A3, NE4049), co-infection (two levels: no, yes), organ (six levels: bladder, left ear, right ear, heart, joint, dorsal skin), and their interactions. Mouse identity was modelled as a random factor. Models that differed with respect to the fixed factor of interest were compared using log-likelihood ratio (LLR) tests to determine statistical significance.

We analysed the prevalence of *B. afzelii* infection in the mouse tissue samples as a function of focal strain, co-infection, and organ. The effect of the three-way interaction between focal strain, co-infection, and organ was not significant (LMM LLR: Δ df = 5, Δ χ^2^ = 0.168, *p = 0.999*). The two-way interactions between co-infection and organ (LMM LLR: Δ df = 5, Δ χ^2^ = 0.518, *p = 0.991*), between co-infection and focal strain (LMM LLR: Δ df = 1, Δ χ^2^ = 0.030, *p = 0.863*), and between focal strain and organ (LMM LLR: Δ df = 5, Δ χ^2^ = 0.734, *p = 0.981*) were not significant. The main effect of co-infection (GLMM LLR: Δ df = 1, Δ χ^2^ = 3.042, *p = 0.081*) and focal strain (GLMM LLR: Δ df = 1, Δ χ^2^ = 3.549, *p = 0.060*) were not significant, whereas organ (GLMM LLR: Δ df = 5, Δ χ^2^ = 22.368, *p < 0.001*) was highly significant.

# Section 5 – Analysis of the total spirochete load of *B. afzelii*

**The total spirochete load per mg of tissue differs among organs:** The spirochete loads estimated by the *flagellin* qPCR in 3 µl of DNA template were corrected to the total DNA extraction elution volume (150 µl), standardized by mg of tissue extracted, and log-10 transformed to improve normality. To test whether there were differences in spirochete load among organs, the log10-transformed spirochete load per mg of tissue was analysed as a linear mixed effects model (LMM). The fixed factor was organ (six levels: bladder, left ear, right ear, heart, joint, skin). Mouse identity was modelled as a random factor.

The effect of organ on the log10-transformed spirochete load per mg of tissue was highly significant (LMM LLR: Δ df = 5, Δ χ^2^ = 934.130, *p < 0.000001*). The mean spirochete loads per mg of tissue were estimated for each of the six organs using the emmeans() function in R, which calculates the estimated marginal means (Table S2). If the heart, which had the lowest spirochete load, is set as the reference, then the spirochete load is 5.2 times higher in the bladder, 5.4 times higher in the ankle joint, 25.7 times higher in the dorsal skin, 122.2 times higher in the right ear, and 147.7 times higher in the left ear (Table S2).

Table S2. The total organ spirochete load (based on *flagellin*-qPCR) per mg of tissue is shown for each of the six organs. The units of the total organ spirochete load are the number of spirochetes per mg of tissue. Shown are the number of tissues that tested positive (N), the mean spirochete load per mg of tissue, the lower limit (LL) and the upper limit (UL) from the 95% confidence interval. Also shown are the log10-transformed estimated marginal means (emmeans), the standard error (SE), and the degrees of freedom (df), which were generated by the emmeans() function in R and used to calculate the mean and 95% confidence interval.

| organ | N | Mean | LL | UL | emmean | SE | df |
| --- | --- | --- | --- | --- | --- | --- | --- |
| bladder | 32 | 185.6 | 139.5 | 246.8 | 2.268 | 0.063 | 148.4 |
| earL | 29 | 5247.9 | 3890.9 | 7078.1 | 3.720 | 0.066 | 151.2 |
| earR | 29 | 4340.7 | 3218.3 | 5854.4 | 3.638 | 0.066 | 151.3 |
| heart | 27 | 35.5 | 26.1 | 48.4 | 1.551 | 0.068 | 153.1 |
| joint | 28 | 192.7 | 142.1 | 261.2 | 2.285 | 0.067 | 152.1 |
| skin | 20 | 914.2 | 638.9 | 1308.1 | 2.961 | 0.079 | 157.5 |

**The total spirochete load per mg of DNA differs among organs:** The spirochete loads estimated by the *flagellin* qPCR in 3 µl of DNA template were corrected to the total spirochete load in 1 µl of template, divided by the DNA concentration (in ng of DNA per µl of DNA extract, as measured by Nanodrop), and multiplied by 1000 so that the units of measurement were the number of spirochetes per mg of DNA. These spirochete loads per mg of DNA were log-10 transformed to improve normality. To test whether there were differences in spirochete load among organs, the log10-transformed spirochete load per mg of DNA was analysed as an LMM. The fixed factor was organ. Mouse identity was modelled as a random factor.

The effect of organ on the log10-transformed spirochete load per mg of DNA was highly significant (Figure S2; LMM LLR: Δ df = 5, Δ χ^2^ = 228.95, *p < 0.000001*). The mean spirochete loads per mg of DNA were estimated for each of the six organs using the emmeans() function in R, which calculates the estimated marginal means (Table S3). If the bladder, which had the lowest spirochete load, is set as the reference, then the spirochete load is 1.2 times higher in the heart, 1.9 times higher in the ankle joint, 2.6 times higher in the dorsal skin, 8.2 times higher in the right ear, and 9.8 times higher in the left ear (Figure S2; Table S3).

Table S3. The total organ spirochete load (based on *flagellin*-qPCR) per mg of DNA is shown for each of the six organs. The units of the total organ spirochete load are the number of spirochetes per mg of DNA. Shown are the number of tissues that tested positive (N), the mean spirochete load per mg of DNA, the lower limit (LL) and the upper limit (UL) from the 95% confidence interval. Also shown are the log10-transformed estimated marginal means (emmeans), the standard error (SE), and the degrees of freedom (df), which were generated by the emmeans() function in R and used to calculate the mean and 95% confidence interval.

| organ | N | Mean | LL | UL | emmean | SE | df |
| --- | --- | --- | --- | --- | --- | --- | --- |
| bladder | 32 | 118.5 | 87.3 | 160.7 | 2.074 | 0.067 | 133.641 |
| earL | 29 | 1161.4 | 844.6 | 1597.1 | 3.065 | 0.070 | 139.181 |
| earR | 29 | 976.4 | 710.1 | 1342.7 | 2.990 | 0.070 | 139.233 |
| heart | 27 | 137.4 | 98.9 | 190.9 | 2.138 | 0.072 | 142.907 |
| joint | 28 | 226.7 | 164.0 | 313.4 | 2.355 | 0.071 | 140.955 |
| skin | 20 | 309.6 | 212.2 | 451.5 | 2.491 | 0.083 | 153.500 |

Figure S2. The spirochete load of *B. afzelii* differs among the six mouse organs. The tissue spirochete load was estimated using a *flagellin* qPCR and was standardized per mg of DNA. The log10-transformed tissue spirochete load per mg of tissue is significantly different among the six mouse organs: bladder, left ear (Ear L), right ear (Ear R), heart, right ankle joint, and the section of dorsal skin where the nymphs had attached. Shown are the means, the 95% confidence intervals, and the individual data points.

**Effect of strain, coinfection, and organ on the total spirochete load per mg of tissue:** The log10-transformed spirochete load per mg of tissue was analysed as an LMM. The fixed factors were focal strain (two levels: Fin-Jyv-A3, NE4049), co-infection (two levels: no, yes), organ (six levels: bladder, left ear, right ear, heart, joint, skin), and their interactions. Mouse identity was modelled as a random factor. Statistical significance of fixed factors was determined by comparing nested models with LLR tests.

The effect of the three-way interaction between strain, co-infection, and organ on the log10-transformed total spirochete load per mg was not significant (LMM LLR: Δ df = 5, Δ χ^2^ = 4.629, *p = 0.463*). The two-way interactions between co-infection and organ (LMM LLR: Δ df = 5, Δ χ^2^ = 5.945, *p = 0.312*), between co-infection and focal strain (LMM LLR: Δ df = 1, Δ χ^2^ = 1.585, *p = 0.208*) and between focal strain and organ LMM LLR: Δ df = 5, Δ χ^2^ = 10.047, *p = 0.074*) were not significant. The main effects of focal strain (LMM LLR: Δ df = 1, Δ χ^2^ = 0.109, *p = 0.741*) and co-infection (LMM LLR: Δ df = 1, Δ χ^2^ = 3.626, *p = 0.057*) were not significant, whereas organ was highly significant (LMM LLR: Δ df = 5, Δ χ^2^ = 305.82, *p < 0.000001*). For each of the 24 combinations of strain, organ, and co-infection, the mean spirochete loads per mg of tissue and their 95% confidence intervals are shown in Table S4.

Table S4. The total organ spirochete load (based on *flagellin*-qPCR) is shown for each of the 24 combinations of strain, organ, and co-infection. The units of the total organ spirochete load are the number of spirochetes per mg of tissue. Shown are the number of tissues that tested positive (N), the geometric means of the total spirochete load and the 95% confidence intervals (95% CI).

| Focal strain | Organ | Co-infection | N |  | Mean total | 95% CI |
| --- | --- | --- | --- | --- | --- | --- |
| Fin-Jyv-A3 | Bladder | no | 9 |  | 157 | 93–265 |
| Fin-Jyv-A3 | Bladder | yes | 7 |  | 221 | 122–401 |
| Fin-Jyv-A3 | Left ear | no | 7 |  | 5,926 | 3,278–10,715 |
| Fin-Jyv-A3 | Left ear | yes | 7 |  | 3,515 | 1,937–6,378 |
| Fin-Jyv-A3 | Right ear | no | 6 |  | 2,533 | 1,336–4,803 |
| Fin-Jyv-A3 | Right ear | yes | 7 |  | 4,957 | 2,732–8,994 |
| Fin-Jyv-A3 | Heart | no | 8 |  | 33 | 19–57 |
| Fin-Jyv-A3 | Heart | yes | 6 |  | 50 | 26–95 |
| Fin-Jyv-A3 | Joint | no | 5 |  | 347 | 172–700 |
| Fin-Jyv-A3 | Joint | yes | 7 |  | 271 | 149–491 |
| Fin-Jyv-A3 | Dorsal skin | no | 5 |  | 1,074 | 533–2,164 |
| Fin-Jyv-A3 | Dorsal skin | yes | 5 |  | 700 | 318–1,540 |
|  |  |  |  |  |  |  |
| NE4049 | Bladder | no | 9 |  | 178 | 106–300 |
| NE4049 | Bladder | yes | 7 |  | 204 | 112–369 |
| NE4049 | Left ear | no | 8 |  | 5,782 | 3,323–10,062 |
| NE4049 | Left ear | yes | 7 |  | 6,541 | 3,605–11,869 |
| NE4049 | Right ear | no | 9 |  | 3,463 | 2,054–5,838 |
| NE4049 | Right ear | yes | 7 |  | 7,450 | 4,106–13,518 |
| NE4049 | Heart | no | 8 |  | 26 | 15–45 |
| NE4049 | Heart | yes | 6 |  | 38 | 19–78 |
| NE4049 | Joint | no | 9 |  | 98 | 58–166 |
| NE4049 | Joint | yes | 7 |  | 214 | 118–389 |
| NE4049 | Dorsal skin | no | 6 |  | 759 | 400–1,439 |
| NE4049 | Dorsal skin | yes | 6 |  | 1324 | 654–2,679 |

# Section 6 – Correlations in spirochete loads among mouse organs

We tested whether the tissue spirochete loads were correlated among the six organs: bladder, left ear, right ear, heart, right ankle joint, and the section of dorsal skin where the nymphs had attached. We investigated the correlations among the organs for the tissue spirochete loads standardized per mg of tissue and for the tissue spirochete loads standardized per mg of DNA. The pairwise correlations are based on the subset of infected tissues (i.e., uninfected tissues are excluded), and the sample sizes for the pairwise correlations therefore range from 17 to 32 mice. We used the *rcorr()* function in the Hmisc package to calculate the correlation matrix and the p-values. We used the *corrplot()* function in the corrplot package to graph the correlation matrix.

**Spirochete load per mg of tissue:** None of the 15 pairwise correlations in total spirochete load standardized per mg of tissue among the six mouse organs were statistically significant (Table S5; Figure S3). There were four pairwise correlations that were positive and that approached statistical significance: bladder versus right ear (*r = 0.347*, *p = 0.065*), left ear versus right ear (*r = 0.342*, *p = 0.088*), left ear versus skin (*r = 0.420*, *p = 0.083*), and right ear versus skin (*r = 0.405*, *p = 0.096*). In summary, there were no significant correlations in total spirochete load standardized per mg of tissue among the six mouse organs (Table S5; Figure S3).

Table S5. Correlation matrix for tissue spirochete load standardized per mg of tissue among the six mouse organs. The pairwise Pearson’s correlation coefficients (r) and their p-values are shown above and below the diagonal. The pairwise correlations are based on the subset of infected tissues (i.e., uninfected tissues are excluded), and the sample sizes for the pairwise correlations therefore range from 17 to 32 mice.

|  | bladder | earL | earR | heart | joint | skin |
| --- | --- | --- | --- | --- | --- | --- |
| bladder | *** | 0.292 | **0.347** | -0.043 | 0.066 | 0.111 |
| earL | 0.124 | *** | **0.342** | -0.341 | -0.157 | **0.420** |
| earR | **0.065** | **0.088** | *** | 0.010 | 0.253 | **0.405** |
| heart | 0.833 | 0.103 | 0.963 | *** | 0.235 | 0.030 |
| joint | 0.737 | 0.443 | 0.213 | 0.280 | *** | -0.094 |
| skin | 0.641 | **0.083** | **0.096** | 0.909 | 0.720 | *** |

**Spirochete load per mg of DNA:** Two of the 15 pairwise correlations in total spirochete load standardized per mg of DNA among the six mouse organs were statistically significant (Table S6; Figure S4): left ear versus skin (*r = 0.541*, *p = 0.021*), and right ear versus skin (*r = 0.723*, *p = 0.001*). There were two pairwise correlations that were positive and that approached statistical significance: bladder versus right ear (*r = 0.362*, *p = 0.054*), and left ear versus right ear (*r = 0.380*, *p = 0.056*). The pattern of pairwise correlations that were significant or almost significant was the same between the spirochete loads standardized per mg of DNA (Table S6; Figure S4) and the spirochete loads standardized per mg of tissue (Table S5; Figure S3). Interestingly, the pairwise correlations were highest for pairs of skin-related organs (e.g., left ear versus skin and right ear versus skin). The fact that the spirochete loads between the left ear and the right ear were not significantly correlated, suggests that there is a lot of random variation in spirochete load among tissues that are functionally the same, but separated in space.

Table S6. Correlation matrix for tissue spirochete load standardized per mg of DNA among the six mouse organs. The pairwise Pearson’s correlation coefficients (r) and their p-values are shown above and below the diagonal. The pairwise correlations are based on the subset of infected tissues (i.e., uninfected tissues are excluded), and the sample sizes for the pairwise correlations therefore range from 17 to 32 mice.

|  | bladder | earL | earR | heart | joint | skin |
| --- | --- | --- | --- | --- | --- | --- |
| bladder | *** | 0.171 | **0.362** | 0.022 | 0.157 | 0.191 |
| earL | 0.375 | *** | **0.380** | 0.126 | 0.024 | **0.541** |
| earR | **0.054** | **0.056** | *** | 0.184 | 0.169 | **0.723** |
| heart | 0.914 | 0.556 | 0.390 | *** | 0.289 | 0.222 |
| joint | 0.424 | 0.906 | 0.410 | 0.180 | *** | -0.208 |
| skin | 0.420 | **0.021** | **0.001** | 0.392 | 0.422 | *** |

Figure S3. Pairwise correlations in spirochete load between the six mouse organs. The tissue spirochete loads were standardized per mg of tissue. The pairwise correlations are based on the subset of infected tissues (i.e., uninfected tissues are excluded), and the sample sizes for the pairwise correlations therefore range from 17 to 32 mice. Positive correlations are shown in blue and negative correlations are shown in red. The size of the circle indicates the magnitude of the correlation coefficient. The numbers inside the circles indicate the statistical significance (p-values) of the pairwise Pearson’s correlation coefficients.

Figure S4. Pairwise correlations in spirochete load between the six mouse organs. The tissue spirochete loads were standardized per mg of DNA. The pairwise correlations are based on the subset of infected tissues (i.e., uninfected tissues are excluded), and the sample sizes for the pairwise correlations therefore range from 17 to 32 mice. Positive correlations are shown in blue and negative correlations are shown in red. The size of the circle indicates the magnitude of the correlation coefficient. The numbers inside the circles indicate the statistical significance (p-values) of the pairwise Pearson’s correlation coefficients.

# Section 7 – The total tissue spirochete load differs among strains

To test whether bacterial abundance differed among the two strains, we analysed the standardized log10-transformed tissue spirochete loads as a function of organ and focal strain for the subset of mice infected with one strain (n = 18). The interaction between focal strain and organ was significant (LMM LLR: Δ df = 5, Δ χ^2^ = 11.85*, p = 0.037*). We therefore used the emmeans function to compare the tissue spirochete load between the two strains separately for each organ. The mean spirochete load in the ankle joints for strain Fin-Jyv-A3 was higher than that of strain NE4049 and this difference was significant (df = 101.5, t = 3.245, *p = 0.0016*). Whereas the differences in spirochete load between strain Fin-Jyv-A3 and strain NE4049 in the bladder (df = 98.0, t = -0.363, *p = 0.717*), the left ear (df = 100.3, t = 0.074, *p = 0.941*), the right ear (df = 100.7, t = -0.653, *p = 0.516*), the heart (df = 99.7, t = 0.590, *p = 0.557*), and the skin (df = 102.3, t = 0.616, *p = 0.540*) were not significant. For each of the 12 combinations of strain and organ, the mean spirochete loads per mg of tissue and their 95% confidence intervals are shown in Table S7.

Table S7. The *B. afzelii* spirochete loads in the mouse tissues are shown separately for the 12 combinations of strain and organ. There are two strains (Fin-Jyv-A3, NE4049) and six organs (bladder, left ear, right ear, heart, right ankle joint, and the section of dorsal skin where the nymphs had attached). The data were calculated for the subset of 18 mice that were singly infected with strain Fin-Jyv-A3 (n = 9) or strain NE4049 (n = 9). The units of the spirochete load are the total number of spirochetes per mg of tissue and the estimates are based on the *flagellin* qPCR. Shown are the number of tissues that tested positive (N), the geometric means of the total spirochete load and the 95% confidence intervals (95% CI).

| Focal strain | Organ | N | Mean | 95% CI |
| --- | --- | --- | --- | --- |
| Fin-Jyv-A3 | Bladder | 9 | 157 | 98–252 |
| Fin-Jyv-A3 | Left ear | 7 | 5,926 | 3,467–10,129 |
| Fin-Jyv-A3 | Right ear | 6 | 2,533 | 1,420–4,520 |
| Fin-Jyv-A3 | Heart | 8 | 33 | 20–54 |
| Fin-Jyv-A3 | Joint | 5 | 347 | 184–655 |
| Fin-Jyv-A3 | Dorsal skin | 5 | 1,074 | 569–2,024 |
|  |  |  |  |  |
| NE4049 | Bladder | 9 | 178 | 111–285 |
| NE4049 | Left ear | 8 | 5,782 | 3,502–9,547 |
| NE4049 | Right ear | 9 | 3,463 | 2,158–5,555 |
| NE4049 | Heart | 8 | 26 | 16–43 |
| NE4049 | Joint | 9 | 98 | 61–158 |
| NE4049 | Dorsal skin | 6 | 759 | 426–1,355 |

# Section 8 – Effect of strain, co-infection, and organ on the strain-specific tissue infection prevalence

The focal strains in experiments 1 and 2 are Fin-Jyv-A3 and NE4049, respectively. In experiment 1, the tissue sample was considered to be infected with Fin-Jyv-A3 if at least two of the three replicate *ospC* A3 qPCR assays tested positive. In experiment 2, the tissue sample was considered to be infected with NE4049 if at least two of the three replicate *ospC* A10 qPCR assays tested positive. The sample size for the analysis is 192 tissue samples. The binomial response variable was whether the focal strain was absent (0) or present (1) in a tissue sample, and it was analysed as a GLMM with binomial errors. The fixed factors were focal strain (two levels: Fin-Jyv-A3, NE4049), co-infection (two levels: no, yes), organ (six levels: bladder, left ear, right ear, heart, joint, dorsal skin), and their interactions. Mouse identity was modelled as a random factor. Models that differed with respect to the fixed factors of interest were compared using log-likelihood ratios (LLR) tests to determine the statistical significance of the fixed factors.

A classic stepwise model simplification approach using LLR tests found that the three-way interaction was not significant and was removed from the analysis (GLMM LLR: Δ df = 5, Δ χ^2^ = 0.502, *p = 0.992*). The two-way interactions between co-infection and organ (GLMM LLR: Δ df = 5, Δ χ^2^ = 1.301, *p = 0.935*), between focal strain and organ (GLMM LLR: Δ df = 5, Δ χ^2^ = 3.580, *p = 0.611*), and between focal strain and co-infection (GLMM LLR: Δ df = 1, Δ χ^2^ = 3.065, *p = 0.080*) were not significant and were removed from the analysis. The tissue infection prevalence of the focal strain in the single strain group (82.4% = 89/108; n = 18 mice) was significantly higher (1.4x) compared to the co-infection group (59.5% = 50/84; n = 14 mice; GLMM LLR: Δ df = 1, Δ χ^2^ = 14.504, *p = 0.0001*). The tissue infection prevalence of strain NE4049 (88.5% = 85/96; n = 16 mice) was significantly higher (1.6x) compared to strain Fin-Jyv-A3 (56.3% = 54/96; n = 16 mice; GLMM LLR: Δ df = 1, Δ χ^2^ = 25.704, *p < 0.000001*), which confirms our previous demonstration that strain NE4049 was more successful at establishing infection in rodent tissues than strain Fin-Jyv-A3. The strain-specific tissue infection prevalence differed significantly among organs (GLMM LLR: Δ df = 5, Δ χ^2^ = 17.830, *p = 0.003*), which confirms our previous demonstration that the *B. afzelii* infection prevalence differs among organs.

As mentioned above, the interaction between focal strain and co-infection was almost statistically significant (GLMM LLR: Δ df = 1, Δ χ^2^ = 3.065, *p = 0.080*). For this reason, the approach taken in the main manuscript was to split the analysis by strain. Co-infection in the rodent host significantly reduced the number of organs infected with strain Fin-Jyv-A3 (GLMM LLR: Δ df = 1, Δ χ^2^ = 15.868, *p = 0.00007*). In contrast, co-infection in the rodent host had no effect on the number of organs infected with strain NE4049 (GLMM LLR: Δ df = 1, Δ χ^2^ = 0.580, *p = 0.446*). Thus, co-infection resulted in competition between strains, which reduced the presence of strain Fin-Jyv-A3, but not strain NE4049, in the host tissues.

For each of the 24 combinations of strain, organ, and co-infection, the strain-specific tissue infection prevalence is shown in Table S8.

Table S8. The strain-specific tissue infection prevalence is shown for each of the 12 unique combinations of focal strain, organ, and co-infection.

| Focal strain | Organ | Co-infection | Prevalence |
| --- | --- | --- | --- |
| Fin-Jyv-A3 | Bladder | No | 100% (9/9) |
| Fin-Jyv-A3 | Bladder | Yes | 57.1% (4/7) |
| Fin-Jyv-A3 | Left ear | No | 77.8% (7/9) |
| Fin-Jyv-A3 | Left ear | Yes | 28.6% (2/7) |
| Fin-Jyv-A3 | Right ear | No | 66.7% (6/9) |
| Fin-Jyv-A3 | Right ear | Yes | 28.6% (2/7) |
| Fin-Jyv-A3 | Heart | No | 88.9% (8/9) |
| Fin-Jyv-A3 | Heart | Yes | 57.1% (4/7) |
| Fin-Jyv-A3 | Joint | No | 55.6% (5/9) |
| Fin-Jyv-A3 | Joint | Yes | 28.6% (2/7) |
| Fin-Jyv-A3 | Skin | No | 55.6% (5/9) |
| Fin-Jyv-A3 | Skin | Yes | 0% (0/7) |
|  |  |  |  |
| NE4049 | Bladder | No | 100% (9/9) |
| NE4049 | Bladder | Yes | 100% (7/7) |
| NE4049 | Left ear | No | 88.9% (8/9) |
| NE4049 | Left ear | Yes | 85.7% (6/7) |
| NE4049 | Right ear | No | 100% (9/9) |
| NE4049 | Right ear | Yes | 100% (7/7) |
| NE4049 | Heart | No | 88.9% (8/9) |
| NE4049 | Heart | Yes | 57.1% (4/7) |
| NE4049 | Joint | No | 100% (9/9) |
| NE4049 | Joint | Yes | 100% (7/7) |
| NE4049 | Skin | No | 66.7% (6/9) |
| NE4049 | Skin | Yes | 71.4% (5/7) |

# Section 9 – Effect of co-infection on the strain-specific tissue infection prevalence in the mice

In this analysis, we ignored the effect of organ and treated each of the 6 tissue samples as an estimate of whether the mouse was infected or not. We analysed the strain-specific prevalence in the mouse tissues as a function of focal strain, co-infection, and their interaction using a generalised linear model (GLM) with binomial errors. The ratio of the residual deviance (22.647) to the residual degrees of freedom (28) was 0.81, indicating that the residuals were not overdispersed. The interaction between co-infection and focal strain was not significant (GLM: Δ df = 1, Δ dev = 2.485, *p = 0.115*) and was removed from the analysis. Both co-infection (GLM: Δ df = 1, Δ dev = 14.405, *p < 0.001*) and focal strain (GLM: Δ df = 1, Δ dev = 28.331, *p < 0.0000001*) were significant. Thus, co-infection resulted in competition between strains, which reduced their presence in the host tissues.

# Section 10 – Effect of strain, co-infection, and organ on the strain-specific tissue spirochete load per mg of tissue

For the subset of infected mouse tissue samples (n = 190), we analysed the strain-specific tissue spirochete load per mg of tissue in mouse tissue samples as a function of focal strain, co-infection, organ, and their interactions. The organ “skin” for strain Fin-Jyv-A3 had to be removed from the analysis because all the tissue samples in the co-infected group tested negative for focal strain Fin-Jyv-A3 in experiment 1. A classic stepwise model simplification approach using LLR tests found that the three-way interaction was significant (LME LLR: Δ df = 4, Δ χ^2^ = 10.633, *p = 0.031*), and the analysis was therefore divided by focal strain.

For strain Fin-Jyv-A3, the interaction between co-infection and organ was significant (Figure S5; LME LLR: Δ df = 4, Δ χ^2^ = 22.324, *p = 0.0002*) and we therefore used the emmeans function to test the effect of co-infection in each organ. In the bladder, co-infection reduced the mean spirochete load of strain Fin-Jyv-A3 by a factor of 4.0 (Figure S5; Table S9; emmeans: df = 58.9, t = 3.109, *p = 0.003*). In the ankle joint, co-infection increased the mean spirochete load of strain Fin-Jyv-A3 by a factor of 5.9 (Figure S5; Table S9; emmeans: df = 67.4, t = -2.353, *p = 0.022*). The contrast between single infection and co-infection was not significant for the other 3 organs (Figure S5; Table S9): left ear (emmeans: df = 65.8, t = 0.447, *p = 0.656*), right ear (emmeans: df = 66.2, t = 1.659, *p = 0.102*), and heart (emmeans: df = 60.3, t = 0.409, *p = 0.684*).

For strain NE4049, the interaction between co-infection and organ was not significant (Figure S6; LME LLR: Δ df = 5, Δ χ^2^ = 3.152, *p = 0.677*). Co-infection was not significant (Figure S6; Table S9; LME LLR: Δ df = 1, Δ χ^2^ = 3.500, *p = 0.061*), but organ had a significant effect on the strain-specific organ spirochete load (Figure S6; Table S9; LME LLR: Δ df = 5, Δ χ^2^ = 600.226, *p < 0.000001*).

For each of the 24 combinations of strain, organ, and co-infection, the mean strain-specific spirochete loads per mg of tissue and their 95% confidence intervals are shown in Table S9. For strain Fin-Jyv-A3, the effect of co-infection on the spirochete load per mg of tissue is shown separately for each of the six organs in Figure S5. For strain NE4049, the effect of co-infection on the spirochete load per mg of tissue is shown separately for each of the six organs in Figure S6.

Table S9. The strain-specific organ spirochete load is shown for each of the 24 unique combinations of focal strain, organ, and co-infection. There are two strains (Fin-Jyv-A3, NE4049), six organs (bladder, left ear, right ear, heart, right ankle joint, and the section of dorsal skin where the nymphs had attached), and two co-infection states (no, yes). The data are shown for the 32 mice that survived to the end of the study. The units of the strain-specific organ spirochete load are the number of spirochetes of the focal strain per mg of tissue. The estimates are based on combining the estimates of total spirochete abundance from the *flagellin* qPCR with the estimates of the strain-specific relative abundance from the strain-specific *ospC* qPCRs. Shown are the number of tissues that tested positive for the focal strain (N), the means and the 95% confidence intervals (95% CI).

| Focal strain | Organ | Co-infection | N | Mean | 95% CI |
| --- | --- | --- | --- | --- | --- |
| Fin-Jyv-A3 | Bladder | no | 9 | 157 | 93–265 |
| Fin-Jyv-A3 | Bladder | yes | 4 | 39 | 18–85 |
| Fin-Jyv-A3 | Left ear | no | 7 | 5,926 | 3,278–10,715 |
| Fin-Jyv-A3 | Left ear | yes | 2 | 4,987 | 1,647–15,101 |
| Fin-Jyv-A3 | Right ear | no | 6 | 2,533 | 1,336–4,803 |
| Fin-Jyv-A3 | Right ear | yes | 2 | 1,151 | 380–3,484 |
| Fin-Jyv-A3 | Heart | no | 8 | 33 | 19–57 |
| Fin-Jyv-A3 | Heart | yes | 4 | 27 | 12–59 |
| Fin-Jyv-A3 | Joint | no | 5 | 347 | 172–700 |
| Fin-Jyv-A3 | Joint | yes | 2 | 2,051 | 677–6,212 |
| Fin-Jyv-A3 | Dorsal skin | no | 5 | 1,074 | 533–2,164 |
| Fin-Jyv-A3 | Dorsal skin | yes | 0 | NA | NA |
|  |  |  |  |  |  |
| NE4049 | Bladder | no | 9 | 178 | 106–300 |
| NE4049 | Bladder | yes | 7 | 170 | 94–307 |
| NE4049 | Left ear | no | 8 | 5,782 | 3,323–10,062 |
| NE4049 | Left ear | yes | 6 | 6,741 | 3,556–12,781 |
| NE4049 | Right ear | no | 9 | 3,463 | 2,054–5,838 |
| NE4049 | Right ear | yes | 7 | 6,506 | 3,598–11,763 |
| NE4049 | Heart | no | 8 | 26 | 15–45 |
| NE4049 | Heart | yes | 4 | 25 | 12–56 |
| NE4049 | Joint | no | 9 | 98 | 58–166 |
| NE4049 | Joint | yes | 7 | 174 | 96–314 |
| NE4049 | Dorsal skin | no | 6 | 759 | 400–1,439 |
| NE4049 | Dorsal skin | yes | 5 | 1,324 | 657–2,668 |

Figure S5. The spirochete load of strain Fin-Jyv-A3 in the mouse tissues depends on organ and co-infection. The spirochete load is measured as the number of spirochetes per mg of tissue. Co-infection significantly decreased the spirochete load of strain Fin-Jyv-A3 in the bladder. Co-infection significantly increased the spirochete load of strain Fin-Jyv-A3 in the ankle joint. The boxplots show the median (black line), 25^th^ and 75^th^ percentiles (edges of the box), minimum and maximum values (whiskers), and outliers (solid circles).

Figure S6. The spirochete load of strain NE4049 in the mouse tissues depends on organ and co-infection. The spirochete load is measured as the number of spirochetes per mg of tissue. Co-infection had no effect on the spirochete load of strain NE4049 in any of the six organs. The boxplots show the median (black line), 25^th^ and 75^th^ percentiles (edges of the box), minimum and maximum values (whiskers), and outliers (solid circles).

# Section 11 – Effect of strain, co-infection, and organ on the strain-specific tissue spirochete load per mg of DNA

For the subset of infected mouse tissue samples (n = 190), we analysed the strain-specific tissue spirochete load per mg of DNA in mouse tissue samples as a function of focal strain, co-infection, organ, and their interactions. The organ “skin” for strain Fin-Jyv-A3 had to be removed from the analysis because all the tissue samples in the co-infected group tested negative for focal strain Fin-Jyv-A3 in experiment 1. A classic stepwise model simplification approach using LLR tests found that the three-way interaction was significant (LME LLR: Δ df = 4, Δ χ^2^ = 13.751, *p = 0.008*), and the analysis was therefore divided by focal strain.

For strain Fin-Jyv-A3, the interaction between co-infection and organ was significant (Figure S7; LME LLR: Δ df = 4, Δ χ^2^ = 22.124, *p = 0.0002*) and we therefore used the parameter estimates to test the effect of co-infection in each organ. Co-infection reduced the mean spirochete load of strain Fin-Jyv-A3 in the bladder (Figure S7; t = 3.149, *p = 0.003*). Co-infection increased the mean spirochete load of strain Fin-Jyv-A3 in the ankle joint (Figure S7; t = 4.431, *p = 0.00007*) and in the heart (Figure S7; t = 2.402, *p = 0.021*). The contrast between single infection and co-infection was not significant for the other 2 organs (Figure S7): left ear (t = 1.264, *p = 0.214*) and right ear (t = 0.369, *p = 0.714*).

For strain NE4049, the interaction between co-infection and organ was not significant (Figure S8; LME LLR: Δ df = 5, Δ χ^2^ = 6.221, *p = 0.285*). Co-infection was not significant (Figure S8; LME LLR: Δ df = 1, Δ χ^2^ = 2.605, *p = 0.107*), but organ had a significant effect on the strain-specific organ spirochete load (Figure S8; LME LLR: Δ df = 5, Δ χ^2^ = 190.834, *p < 0.000001*).

For strain Fin-Jyv-A3, the effect of co-infection on the spirochete load per mg of DNA is shown separately for each of the six organs in Figure S7. For strain NE4049, the effect of co-infection on the spirochete load per mg of DNA is shown separately for each of the six organs in Figure S8.

Figure S7. The spirochete load of strain Fin-Jyv-A3 in the mouse tissues depends on organ and co-infection. The spirochete load is measured as the number of spirochetes per mg of DNA. Co-infection significantly decreased the spirochete load of strain Fin-Jyv-A3 in the bladder. Co-infection significantly increased the spirochete load of strain Fin-Jyv-A3 in the ankle joint and the heart. The boxplots show the median (black line), 25^th^ and 75^th^ percentiles (edges of the box), minimum and maximum values (whiskers), and outliers (solid circles).

Figure S8. The spirochete load of strain NE4049 in the mouse tissues depends on organ and co-infection. The spirochete load is measured as the number of spirochetes per mg of DNA. Co-infection had no effect on the spirochete load of strain NE4049 in any of the six organs. The boxplots show the median (black line), 25^th^ and 75^th^ percentiles (edges of the box), minimum and maximum values (whiskers), and outliers (solid circles).

# Section 12 – Relationship between host-to-tick transmission of the focal strain and the number of organs infected by the focal strain

We wanted to test whether host-to-tick transmission of the focal strain was dependent on the infection status of the focal strain in any particular organ. For each mouse, we summed the infection status of the focal strain across all six organs. We used a GLMER with binomial errors to model host-to-tick transmission of the focal strain as a function of the number of organs infected with the focal strain. The number of organs infected with the focal strain had a significant and positive effect on the host-to-tick transmission of the focal strain (Figure S9; GLMM LLR: Δ df = 1, Δ χ^2^ = 45.124, *p < 0.000001*). However, the ratio of the residual deviance to the residual degrees of freedom (123.02/30 = 4.101) suggest that the data were overdispersed and we therefore re-analysed the data with a quasibinomial distribution. After correction for overdispersion, the positive relationship between the number of organs infected with the focal strain and the host-to-tick transmission of the focal strain remained statistically significant (GLMM LLR: Δ df = 1, Δ χ^2^ = 12.279, *p = 0.0005*).

Figure S9. Host-to-tick transmission of the focal strain depends on the number of organs that are infected with the focal strain. Strain Fin-Jyv-A3 and strain NE4049 are shown in blue and red symbols, respectively. Mice in the single strain groups and the co-infected groups are shown with solid and empty symbols, respectively. The black line shows the line of best fit that was estimated from a GLM.

# Section 13 – Relationship between host-to-tick transmission of the focal strain and the mean organ spirochete load of the focal strain

We wanted to test whether host-to-tick transmission of the focal strain was dependent on the spirochete load of the focal strain in any particular organ. For each mouse, we averaged the log10-transformed spirochete load of the focal strain across the subset of infected organs. We used a GLMER with binomial errors to model the host-to-tick transmission of the focal strain as a function of the mean log10-transformed spirochete load of the focal strain in the organs (hereafter referred to as organ spirochete load for convenience). The mean organ spirochete load of the focal strain had a significant positive effect on the host-to-tick transmission of the focal strain (Figure S10; GLMM LLR: Δ df = 1, Δ χ^2^ = 36.165, *p < 0.000001*). However, the ratio of the residual deviance to the residual degrees of freedom (131.98/30 = 4.399) suggest that the data were overdispersed and we therefore re-analysed the data with a quasibinomial distribution. After correction for overdispersion, the positive relationship between the log10-transformed mean spirochete load of the focal strain and the host-to-tick transmission of the focal strain remained statistically significant (GLMM LLR: Δ df = 1, Δ χ^2^ = 9.090, *p = 0.003*).

Figure S10. Host-to-tick transmission of the focal strain depends on the mean spirochete load of the focal strain in the mouse tissues. For each mouse, the spirochete load of the focal strain was averaged over the subset of infected organs (the size of each circle indicates the number of infected organs). Strain Fin-Jyv-A3 and strain NE4049 are shown in blue and red symbols, respectively. Mice in the single strain groups and the co-infected groups are shown with solid and empty symbols, respectively. The black line shows the line of best fit that was estimated from a GLM.

As we have shown previously, the mean spirochete load differs dramatically among organs. In general, the spirochete load is higher for external organs (e.g., left ear, right ear, and dorsal skin) compared to internal organs (bladder, heart, ankle joint). In Figure S10, the mean spirochete load is biased by the subset of organs that are infected. Mean spirochete loads based on external organs will be higher than means based on internal organs. To avoid this problem of bias, we transformed the spirochete loads to z-scores for each organ (and excluded the uninfected tissue samples). Thus, each organ had a mean spirochete load of zero and a standard deviation of 1. For each mouse, we then calculated the spirochete load of the focal strain across the subset of infected organs. We refer to this variable as the mean organ-adjusted spirochete load.

The mean organ-adjusted spirochete load of the focal strain had a significant positive effect on the host-to-tick transmission of the focal strain (Figure S11; GLMM LLR: Δ df = 1, Δ χ^2^ = 17.218, *p = 0.00003*). However, the ratio of the residual deviance to the residual degrees of freedom (150.93/30 = 5.031) suggest that the data were overdispersed and we therefore re-analysed the data with a quasibinomial distribution. After correction for overdispersion, the positive relationship between the mean organ-adjusted spirochete load of the focal strain and the host-to-tick transmission of the focal strain was almost statistically significant (GLMM LLR: Δ df = 1, Δ χ^2^ = 3.827, *p = 0.050*).

Figure S11. Host-to-tick transmission of the focal strain depends on the mean organ-adjusted spirochete load of the focal strain in the mouse tissues. To correct for differences in spirochete load among organs, the spirochete loads were transformed to z-scores for each organ (i.e. each organ had a mean of 0, and a standard deviation of 1). For each mouse, the organ-adjusted spirochete loads of the focal strain were averaged over the subset of infected organs (the size of each circle indicates the number of infected organs). Strain Fin-Jyv-A3 and strain NE4049 are shown in blue and red symbols, respectively. Mice in the single strain groups and the co-infected groups are shown with solid and empty symbols, respectively. The black line shows the line of best fit that was estimated from a GLM.

# Section 14 – References

1. Honeder S, Drskova E, Danklmaier A, Vavruskova Z, Martin S, Grubhoffer L, et al. Now you see mee: Transformation of infectious *Borrelia afzelii* with fluoresccent reporters *Submitted: Pathogens*. 2020.

2. Genné D, Sarr A, Gomez-Chamorro A, Durand J, Cayol C, Rais O, et al. Competition between strains of *Borrelia afzelii* inside the rodent host and the tick vector. *Proc R Soc B*. 2018;285(1890):20181804.

3. Genné D, Sarr A, Rais O, Voordouw MJ. Competition between strains of *Borrelia afzelii* in immature *Ixodes ricinus* ticks is not affected by season. *Front Cell Infect Microbiol*. 2019;9(431).

4. Schwaiger M, Peter O, Cassinotti P. Routine diagnosis of *Borrelia burgdorferi* (sensu lato) infections using a real‐time PCR assay. *Clin Microbiol Infect*. 2001;7(9):461-9.

5. Bunikis J, Garpmo U, Tsao J, Berglund J, Fish D, Barbour AG. Sequence typing reveals extensive strain diversity of the Lyme borreliosis agents *Borrelia burgdorferi* in North America and *Borrelia afzelii* in Europe. *Microbiology*. 2004;150(6):1741-55.
